# The role of lipids in regulating macrophage antifungal immunity

**DOI:** 10.1128/mbio.03057-23

**Published:** 2024-08-29

**Authors:** Sophie Rouvray, Rebecca A. Drummond

**Affiliations:** 1 Institute of Immunology and Immunotherapy, University of Birmingham, Birmingham, United Kingdom; Instituto Carlos Chagas, Curitiba, Brazil

**Keywords:** cholesterol, macrophages, *Cryptococcus neoformans*, meningitis

## Abstract

Macrophages are critical components of the antifungal immune response. Disturbance in the number or function of these innate immune cells can significantly increase susceptibility to invasive fungal infections. Pathogenic fungi cause billions of infections every year and have an unmet clinical need, with many infections associated with unacceptably high mortality rates that primarily affect vulnerable patients with underlying immune defects. Lipid metabolism has been increasingly appreciated to significantly influence macrophage function, particularly of macrophages residing in lipid-rich organs, such as the brain, or macrophages specialized at clearing dead cells including alveolar macrophages in the lungs. In this review, we provide an overview of macrophage lipid metabolism, and discuss how lipid recycling and dysregulation affect key macrophage functions relevant for antifungal immunity including phagocytosis, functional polarization, and inflammasome activation. We focus on the fungal pathogen *Cryptococcus neoformans*, as this is the most common cause of death from fungal infection in humans and because several lines of evidence have already linked lipid metabolism in the regulation of *C. neoformans* and macrophage interactions.

## INTRODUCTION

Lipids are a key structural component of every cell membrane throughout the body. In addition, lipids are essential for the synthesis, transport and absorption of hormones, the maintenance of energy homeostasis, and signaling for both intracellular and extracellular cell–cell communication (1). Tight regulation of lipid homeostasis is therefore essential for proper functioning of leukocytes and is integral to the effective control of immune responses. For example, macrophages remodel their “lipidome” following exposure to inflammatory signals and/or phagocytosis of microbes (2). Some lipid species are crucial for mitochondrial respiration, a process that underlies metabolic rewiring in macrophages and their functional differentiation into pro- and anti-inflammatory phenotypes. However, excess lipids, particularly cholesterol, can be highly toxic to mammalian cells, impairing their function by causing cellular stress and driving pro-apoptotic pathways (3). Therefore, macrophages residing in lipid-rich organs, such as the brain, liver, and adipose tissue, have evolved sophisticated mechanisms to counteract potential lipid-mediated toxicity (4).

The most lipid-rich organ in the human body is the brain, with lipids accounting for approximately 50% of its dry weight (5). Abnormal levels of lipids in the central nervous system (CNS) can severely impact neurological development and function, and dysregulation of lipid metabolism is a defining feature of several neurodegenerative diseases including Alzheimer’s, Huntington’s, and Parkinson’s disease (6). In particular, many of these diseases cause the formation of lipid-laden or inflammatory brain-resident macrophages called microglia, which have enhanced lipid metabolism and are associated with disease progression (7, 8).

While there has been much progress in understanding lipid dysregulation in the brain and particularly within microglia during neurodegenerative disorders, much less is understood about how lipid homeostasis and remodeling are regulated during CNS infection. Brain infections and meningitis may be caused by viruses, bacteria, parasites, and fungi, with susceptibility in humans enhanced by specific defects in host immunity, extremes of age, and/or brain injury. One of the most poorly understood group of brain infections are those caused by fungal pathogens. Although typically rarer than other types of infections, fungal meningitis causes up to approximately 150,000 deaths every year and is associated with limited treatment options (9). The majority of fungal brain infections in humans are caused by the fungus *Cryptococcus neoformans*, which primarily infects patients with advanced HIV infection in sub-Saharan Africa (10). Brain-specific immune responses to this fungal pathogen are poorly understood; however, some recent studies have indicated that lipids may play an integral role in macrophage interactions with this fungus, which are a critical determinant of infection outcome.

In this review, we will discuss how lipid metabolism influences macrophage function, particularly during infection and in the brain. We highlight recent studies that have demonstrated critical roles for lipid droplets and cholesterol metabolism in influencing the inflammatory phenotype of macrophages, using studies from other fields, to demonstrate how lessons may be applied to the context of fungal infection, with the aim of inspiring new questions and future directions for the fungal immunology and medical mycology fields.

## CRYPTOCOCCAL MENINGITIS: CLINICAL IMPACT, PATHOGENESIS, AND IMMUNITY

Invasive fungal infections cause over 1.5 million deaths worldwide every year with mortality rates ranging between 20% and 90% (9). Invasive fungal infections predominantly affect vulnerable patients with compromised immune systems, such as those with HIV/AIDS, or those on immunosuppressant treatments associated with chemotherapy or solid organ transplantation (9). The World Health Organization published its first priority fungal pathogen list in 2022, highlighting the fungal species and infections that urgently require more research and input around health policy (11). In the critical priority group, there were several fungal species listed that all cause brain infections to varying degrees. These included *C. neoformans*, the most common cause of fungal brain infections in humans, as well as *Candida albicans* and *Aspergillus fumigatus*. The latter two species are rare causes of severe CNS disease in patients with specific immune deficiencies, such as CARD9 deficiency (*Candida*), and following treatment with ibrutinib (*Aspergillus*) (12). Other fungal pathogens that may cause CNS disease include *Histoplasma capsulatum*, *Coccidioides immitis*, and *Blastomyces dermatitidis* (13). These dimorphic fungal pathogens are endemic to regions in the United States, and account for almost a third of community-acquired pneumonia in these areas. The diagnosis and treatment for dimorphic fungal infections are often delayed since their clinical presentations typically overlap with bacterial pneumonias, resulting in misuse of antibiotics (14). Generally, treatment of fungal CNS infections is challenging, and as a result, mortality rates are typically higher for this group of infections than for other types of invasive fungal disease. Many antifungal drugs do not efficiently cross the blood–brain barrier (BBB), which limits their efficacy, and surgical debridement of infected tissue is not always possible in the brain (15). Moreover, there are socioeconomic and political challenges that limit access to the most effective treatments for fungal meningitis in the countries that have the highest burden of disease, and therefore, many deaths caused by CNS fungal infection are thought to be preventable (16). This particularly applies to *C. neoformans* infection, which is the most common cause of fungal brain infection. Due to its high frequency and mortality rate, we will focus on *C. neoformans* infections for this review. However, it is likely that many of the principles we will discuss will also apply to other fungal pathogens as well.

Cryptococcal meningitis is a fatal brain infection affecting approximately 200,000 people globally each year, with approximately 147,000 deaths (17). Over 70% of these deaths occur in low-income countries, and many are considered to be preventable (18). The major risk factor promoting susceptibility to cryptococcal infection is a failure of CD4 T-cell immunity, usually caused by advanced HIV infection, but is also observed in non-HIV patients with other types of immunosuppression affecting lymphocyte responses (19). *C. neoformans* can colonize and infect various tissues and organs throughout the body; however, the clinical presentations of disease are typically associated with CNS infection. Cryptococcal meningitis initially presents with neurological symptoms including headache, nausea, stiff neck, confusion, sensitivity to light, and lethargy (20). In immunocompetent patients, these symptoms may develop over a period of 6–12 weeks. However, in immunocompromised patients, symptoms rapidly emerge within 1–2 weeks, resulting in death if left untreated (20).

*C. neoformans* exists ubiquitously in the environment and is commonly found in bird droppings, soil, and a variety of trees species (21). Infections arise following the inhalation of *C. neoformans* spores and/or desiccated yeast, which colonize the alveolar spaces of the lungs. If pulmonary immunity fails to clear these spores, they may germinate into yeast cells and escape the lungs, disseminating to other tissues but particularly target the CNS (21, 22). In some patients, it is thought that granulomas may form around *C. neoformans* spores and infection controlled by these structures unless immune-suppression occurs, resulting in a breakdown of the granuloma and “escape” of the fungus to distant sites. This theory has been backed up by experimental evidence in mouse models, which demonstrated that disruption to granuloma structures by blocking protective immunity mediated by lymphocytes resulted in granuloma disorganization and fungal dissemination (23).

Protective immunity against *C. neoformans* relies on proper activation of macrophages, which results from crosstalk with Th1-polarized CD4 T cells. CD4 T cells that produce interferon gamma (IFNγ) (Th1) are protective against *C. neoformans* because IFNγ instructs macrophages to express antifungal enzymes (such as inducible nitric oxide synthase [iNOS]) and promote maturation of phagolysosomes, which aid in fungal killing. Indeed, there are strong correlations observed in cryptococcal meningitis patients between IFNγ levels and fungal burdens in the cerebrospinal fluid, where greater IFNγ levels typically correlate with reduced fungal burdens and better clinical outcomes (24, 25). The absence of IFNγ-mediated activation of macrophages is associated with an anti-inflammatory activation profile in infected patients, resulting in poor fungal killing and the development of intracellular infection reservoirs (26). It has been well documented since the 1970s that *C. neoformans* yeast phagocytosed by macrophages (that have not had prior IFNγ-mediated activation) have the ability to survive inside macrophage phagosomes and, in fact, can replicate at a higher rate within macrophages in comparison to the extracellular environment, at least *in vitro* (27–29). This is in part due to the striking ability of *C. neoformans* to modulate the intracellular environment of phagosomes and the functional phenotype of macrophages. For example, while acidification of the phagosome normally aids in killing of phagocytosed pathogens, *C. neoformans* was found to have a higher growth rate within acidic conditions (pH 5) (30) related to the buffering capacity of the polysaccharide capsule that surrounds the *C. neoformans* cell wall (31). *C. neoformans* also has mechanisms to damage the membrane of the phagolysosome, preventing full maturation (32). Finally, *C. neoformans* was recently shown to secrete a small protein called CLP-1, which drives expression of the enzyme arginase in macrophages (33). Arginase competes with the antifungal enzyme iNOS, and therefore, CLP-1 secretion by the fungus mediates its intracellular survival within macrophages and promotes virulence of the pathogen (33). The growth of *C. neoformans* within macrophages has been shown to be a relevant factor in determining its pathogenicity in mouse models of infection and a driver of population heterogeneity. For example, *C. neoformans* yeast residing in brain-resident macrophages were found to be protected against copper starvation, whereas yeast that were extracellularly growing in this tissue significantly upregulated copper transporters, resulting in the formation of a heterogeneous population of yeast in the infected brain (34). In addition, *C. neoformans* residence within myeloid cells has been shown to promote dissemination from the lungs within infected monocytes (Trojan Horse method) (35), which has been observed using both *in vitro* models and *in vivo* infection studies (35, 36). For example, exposing mice to infected monocytes has been shown to result in earlier brain dissemination (36), while monocyte depletion can help reduce brain dissemination from the lungs, although this protective effect is dependent on timing of depletion (37).

**Fig 1:**
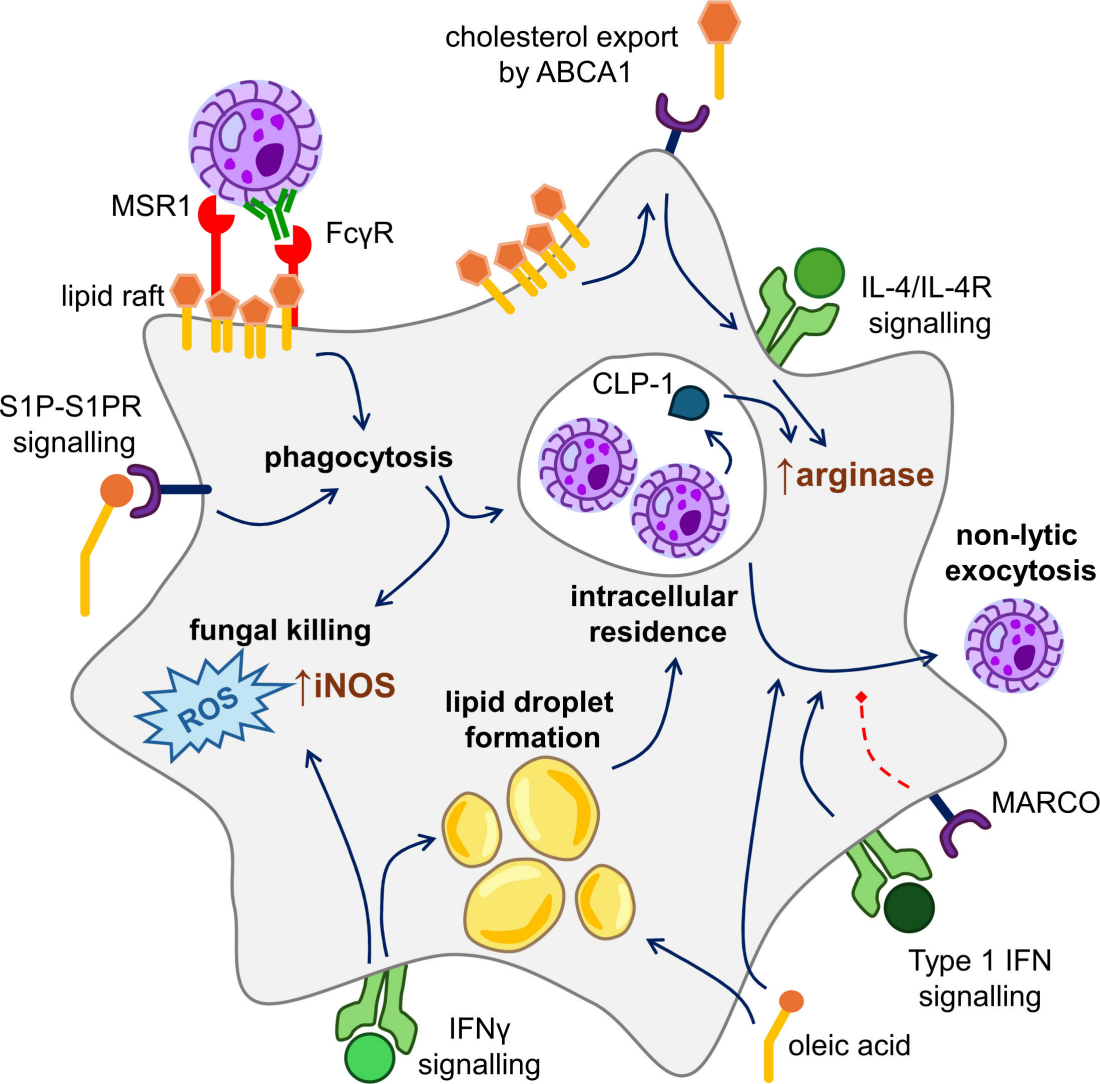
Overview of *Cryptococcus neoformans* interactions with macrophages and the role of lipids. *C. neoformans* is recognized by innate receptors expressed by macrophages, primarily MSR1 (unopsonized yeast) and FcγR (opsonized yeast). These receptors are concentrated within lipid rafts in the macrophage membrane, which contain high concentrations of cholesterol and sphingomyelin. These lipids support downstream signaling to drive phagocytosis. Soluble lipids, such as S1P, may also boost phagocytosis via S1P receptor signaling (see text for details). Following phagocytosis, *C. neoformans* may be killed via reactive oxygen species (ROS) and iNOS activity, or reside with phagosomes, depending on the activation status of the macrophage. Interferon gamma (IFNγ) signaling drives expression of iNOS and supports fungal killing and also drives the formation of cholesterol-rich lipid droplets, which can act as immune signaling hubs but may also feed intracellular yeast. In contrast, interleukin 4 (IL-4) signaling drives arginase expression, which supports intracellular fungal residence. CLP-1, secreted by the fungus, can also boost arginase expression. In the context of cancer, cholesterol export and depletion of lipid rafts supports IL-4 signaling and arginase expression In macrophages. In some cases, *C. neoformans* is expelled from the macrophage in a process called non-lytic exocytosis. This process is negatively regulated by the receptor macrophage receptor with collagenous domain (MARCO) and positively regulated by type 1 IFN signaling as well as exposure to some fatty acids (e.g., oleic acid). Type 1 IFN signaling and supplementation with oleic acid may also affect lipid homeostasis within the macrophages, for example, by contributing toward the formation of lipid droplets (see main text for further details).

Intracellular infection of macrophages and pathogenicity of *C. neoformans* are influenced by host lipids (Fig. 1). For example, cholesterol and sphingomyelin are required for the phagocytosis of fungal cells by macrophages, since the depletion of these lipids from host cell membranes reduced phagocytosis of *C. neoformans* yeast (38). This, in turn, affects the development of intracellular infections of macrophages, but host lipids may also affect the subsequent intracellular survival of the fungus. For instance, lipid droplets within macrophages have been shown to accumulate on the surface of the phagolysosome, providing an accessible source of lipids to be utilized in carbon metabolism pathways that *C. neoformans* uses to generate energy (39). Oleic acid, a fatty acid that undergoes esterification and storage in lipid droplets, was shown to enhance the fungal replication rate of both intracellular and extracellular *C. neoformans*, confirming the ability of the fungus to access and utilize sources, fatty acids from lipid droplets within macrophages (39). Foamy macrophages, which have increased lipid content and dysregulated immune function, are often observed in histological analysis of *C. neoformans*-infected tissues in mouse models of the infection (40–42), as well as in human autopsy samples (43, 44). More broadly, lipid peroxidation has been observed in a rabbit *C. neoformans* infection model (45), and some clinical case studies have identified dysregulated levels of circulating cholesterol in cryptococcal meningitis patients (46). Indeed, a genome-wide association study of patients with HIV-associated cryptococcal meningitis identified single-nucleotide polymorphisms in genes regulating macrophage activation and cholesterol metabolism (47).

In addition to the influence of host lipids, *C. neoformans* itself encodes multiple enzymes and transporters that can synthesize, degrade, or uptake lipids, which, in turn, may affect lipid-dependent host immune mechanisms and/or contribute toward the immune-modulating phenotypes observed with this fungus. For example, screening studies that aimed to identify *C. neoformans* mutants with growth defects in cerebrospinal fluid or serum found that deletion of phosphatidylinositol 4-kinase (PIK1), an enzyme that synthesizes phosphatidylinositol-4-phosphate, which regulates lipid transport in the Golgi, had a significant reduction in the ability to reside intracellularly within macrophages and had a reduced virulence potential in both murine and rabbit infection models (48, 49). Deletion of phospholipase B, an enzyme that breaks down lipid chains, also reduces *C. neoformans* brain invasion and residence in microglia during *in vitro* and mouse infection models (50). Comparison of lipid abundance in *Cryptococcus* strains also reveals marked variation in the abundance of different lipid species between isolates, particularly sphingolipids (51). However, whether these variations contribute toward the observed heterogeneity in virulence profiles of clinical isolates is not clear.

Although host lipids have the potential to significantly influence interactions between macrophages and *C. neoformans*, our current understanding of the lipid species and regulation of lipid remodeling during fungal infection is still in its infancy. The next parts of this review will provide an overview of how lipids are remodeled and used in promoting macrophage functions that are critical for antifungal defense, highlighting key areas for developing future research questions.

Box 1. **An introduction to lipid species and their localization in macrophages**

In mammalian cells, different organelles have specific lipid compositions that can vary depending on the cell type. For example, the plasma membrane is primarily comprised of glycerophospholipids (GPLs), sphingolipids, and sterols, with cholesterol making up to a third of the lipid bilayer (52). Cholesterol content may vary across the plasma membrane, which helps regulate membrane fluidity. High concentrations of cholesterol in the membrane form lipid rafts, which are micro-domains that are essential for endocytosis/exocytosis as well as cell signaling and communication (53).

While lipid composition of plasma membranes can be dynamic and change with cellular responses to environmental stimuli, lipid compositions in the mitochondria are typically more stable. Mitochondria are membrane-bound organelles found within nearly all eukaryotic cells that are involved in energy (ATP) production (54). Mitochondria are highly dynamic and remodel their shape and activity in response to inflammation and infection. This, in part, supports metabolic rewiring of macrophages, which supports their differentiation toward phenotypes that support fungal killing (e.g., IFNγ-activated iNOS expressing macrophages) or promote intracellular fungal infection (e.g., arginase expressing macrophages). Additionally, mitochondria produce reactive oxygen species (ROS), which have antifungal killing properties but also help immune cells to regulate their proliferation, polarization, and signaling (55, 56).

Mitochondria have both inner and outer membranes, which aid the flow of electrons in the electron transport chain. Mitochondrial membranes are primarily made from the phospholipids phosphatidylcholine (PC) and phosphatidylethanolamine (PE), in addition to mitochondria-specific cardiolipin and relatively small amounts of phosphatidylserine (PS), phosphatic acid (PA) and phosphatidylglycerol (PG) (53, 57). Disruption to the lipid composition of mitochondria is typically associated with dysfunction and has been linked with the progression of neurodegenerative diseases (58). For example, the major protein in the pathogenesis of Parkinson’s disease, alpha-Synuclein (α-Syn), is known to closely interact with cardiolipin in mitochondrial membranes. This interaction allows α-Syn to impair the membrane integrity of mitochondria and cause mitochondrial dysfunction, a major contributor to the etiology of Parkinson’s disease. However, in some situations, changes to mitochondrial lipid remodeling can be associated with protection against diseases where mitochondrial dysfunction is a feature, such as cancer. For example, cardiolipin is synthesized by cardiolipin synthase 1 (CRLS1), and an abnormally high expression of CRLS1 has been shown to be associated with improved survival of patients with non-small cell lung cancer and lung adenocarcinoma, highlighting a potential link between the regulation of mitochondrial lipids and tumor suppression (59).

Within the macrophage, the balance of lipid species and regulation of their abundance must be tightly controlled to avoid toxicity. For example, cholesterol is a required element of plasma membranes and cell signaling pathways, and therefore, macrophages have several receptors that allow them to acquire cholesterol and other lipids/lipoproteins, including scavenger receptors and CD36. Cholesterol that is taken up by the cell is then recycled through a series of enzymatic reactions, eventually being stored in small organelles called lipid droplets. Lipid droplets primarily contain glycerophospholipids and cholesterol esters, although their composition can vary widely between cell type. For example, lipid droplets from brain macrophages (microglia) contain mostly glycerophospholipids (8), whereas cholesterol esters are highly abundant in macrophages isolated from atherosclerotic lesions (60). Lipid droplets are the main site of cholesterol hydrolysis, a chemical reaction that enables cholesterol to be exported from the cell via efflux transporter ABCA1 (60). Disruption to these lipid recycling pathways can cause a build-up of lipid droplets, which is associated with cellular stress (particularly on the endoplasmic reticulum) and activation of pro-apoptotic pathways (61). Indeed, disruption of the transport of cholesterol and other lipids through cells often results in significant disease and is typically accompanied by macrophage dysfunction. For example, atherosclerosis is a condition characterized by high levels of circulating cholesterol and promotes cardiovascular disease. Macrophages from these patients have significant build-up of lipids and lipid droplets in their cytoplasm, affecting their inflammatory functions and contributing toward vessel damage (4). Similarly, brain macrophages from patients and mice with *NPC1* mutations, which cause the lysosomal storage disorder Niemann Pick C, have dysregulation of cholesterol trafficking and a build-up of cholesterol in brain macrophages, impairing their functions and contributing toward immune-driven pathology, particularly in the CNS (62).

## MACROPHAGE LIPID METABOLISM DURING ANTI-FUNGAL IMMUNE RESPONSES

As outlined above, macrophage responses to *C. neoformans* are a critical determinant of infection outcome, and there is some initial evidence indicating that lipids may influence this (Fig. 1). In this section, we discuss how lipids contribute toward the major functions of macrophages relevant for antifungal immune responses, indicating the areas that require further research.

### Phagocytosis

Uptake of *C. neoformans* yeast by macrophages is a critical first step toward fungal killing and clearance of infection. However, phagocytosis may also lead to the development of intracellular infection of macrophages and evasion of the fungus from the host immune system. Phagocytosis is therefore associated with both protective and non-protective outcomes.

Lipid rafts in the cell membrane are required for cell signaling and initiation of phagocytosis following innate recognition of yeast via pattern recognition receptors (PRRs). Important antifungal PRRs include the C-type lectin receptors (CLRs), such as Dectin-1, and the Toll-like receptors (TLRs), such as TLR-2 and TLR-4 (63). *C. neoformans* is predominantly recognized by the CLR CD206 (also called the mannose receptor) (64), and several members of the TLR family, which bind components within the fungal capsule. CLRs that bind elements of the fungal cell wall (e.g., Dectin-1, Dectin-2) are typically not required for *C. neoformans* recognition as the capsule shields the cell wall from these receptors (65). Recent studies have highlighted at complex crosstalk that exists between these receptors, which regulates phagocytosis of *C. neoformans* yeast cells and/or is involved in the recognition of secreted immune-modulating proteins produced by *C. neoformans* that promote its virulence. For example, TLR-4 can bind to carbohydrates within the *C. neoformans* capsule, but it also negatively regulates expression of the macrophage scavenger receptor (MSR1, CD204) which mediates uptake of unopsonized *C. neoformans* via multi-protein signaling complexes involving the FcγR receptor and ERK kinases (66). TLR-4 also recognizes and binds CLP-1, a driver of arginase expression and intracellular fungal infection in macrophages (33).

All of these fungal-recognizing PRRs are localized to lipid rafts (67), which contain a high concentration of cholesterol and the lipid sphingomyelin. Both lipids are essential for *C. neoformans* uptake, likely by promoting the formation of lipid rafts, which bring PRRs (particularly FcγR) together for effective downstream signaling (38), and activation of cytoskeleton remodeling pathways that are required to support phagocytosis, as well as providing membrane fluidity that is needed for engulfment. In addition to cholesterol, lipid rafts also have a high concentration of a type of lipid called plasmalogens. Plasmalogens are a specialized type of glycerophospholipid found ubiquitously in lipid rafts and play a key role in phagocytosis. Plasmalogen-deficient macrophages (RAW246.7 macrophage cell line) had a defective uptake of zymosan, a carbohydrate mixture that is recognized by Dectin-1 and TLR-2, which was associated with reduced size and number of lipid rafts within the plasma membrane (68–70). Plasmalogens may also mediate the inflammatory function of macrophages, particularly microglia. Microglia are brain-resident macrophages that phagocytose *C. neoformans* rapidly upon invasion (71), yet are susceptible to intracellular infection and do not provide protection against this infection (34). Treatment of pre-activated inflammatory microglia with plasmalogens decreased their production of nitric oxide and pro-inflammatory cytokines (72). These data indicate that these specialized lipids may have profound effects on microglia function and their ability to kill *C. neoformans*, although the specific mechanisms by which plasmalogens achieve these anti-inflammatory effects are still unclear, and no work has yet been done to determine the involvement of plasmalogens in uptake of yeast by macrophages. Moreover, it is also not clear if the anti-inflammatory effects of plasmalogens observed in microglia would apply to other macrophage subsets, such as monocyte-derived inflammatory macrophages or meningeal border macrophages in the brain, which can have significantly different uptake rates of *C. neoformans* and may therefore be differentially affected by the immune-modulating effects of different lipid species.

In addition to the influence of lipids that form cellular structures (e.g., plasma membrane), there are also signaling lipids that influence macrophage function and phagocytosis. For example, sphingosine-1-phosphate (S1P) is a signaling lipid that exerts its action via binding to S1P receptors, which are highly expressed by macrophages and immune cells. S1P was shown to boost uptake of *C. neoformans* by alveolar macrophages via the S1P receptor 2 (S1PR2) in a mechanism that involved FcRγ receptors since macrophages that lacked expression of S1PR2 had significantly reduced expression of these opsonic receptors (73), which, as outlined above, are important mediators of *C. neoformans* uptake via lipid rafts in the membrane. These observations help explain the increased incidence of *C. neoformans* infections in patients treated with S1P analogs (e.g., FTY720, a treatment for multiple sclerosis), since these drugs block S1P receptors (particularly S1PR3) and reduce macrophage uptake of *C. neoformans* and subsequent production of reactive oxygen species (23), an important fungal-killing mechanism (see below).

### Non-lytic exocytosis (vomocytosis)

Macrophages that have phagocytosed microbes but are unable to kill them have been observed to expel their phagocytic cargo in a process called non-lytic exocytosis (also called vomocytosis). Non-lytic exocytosis has been observed in macrophages interacting with *C. neoformans* and is a process hypothesized to promote infection and fungal escape from macrophages (74). The molecular mechanisms regulating non-lytic exocytosis are currently unclear with only a few enzymes and receptors identified as playing essential roles in this process. Inhibition of ERK5, a member of the MAPK family, promoted a pro-inflammatory polarization in macrophages and resulted in enhanced vomocytosis of live *C. neoformans* (75), while the membrane-binding protein annexin A2 appears to promote both the phagocytosis and vomocytosis of *C. neoformans* (76). To date, the most striking phenotype in non-lytic exocytosis of *C. neoformans* has been observed in macrophages lacking expression of the scavenger receptor macrophage receptor with collagenous domain (MARCO). MARCO-deficient macrophages displayed an almost 100% rate of exocytosis of internalized *C. neoformans*, suggesting that MARCO is a predominant negative regulator of this process in the context of *C. neoformans* infection (77). On the fungal side, it is not clear whether there are fungal-intrinsic factors that select yeast cells for non-lytic exocytosis by macrophages. This is an important aspect to consider, particularly considering that heterogeneity in the *C. neoformans* population within the infected brain has been recently observed (34).

Since non-lytic exocytosis is intimately linked with phagocytosis and plasma membrane function, lipids play an integral regulatory role in this process. Exogenous treatment with the fatty acid lipid oleic acid of cultured macrophages significantly increased rates of non-lytic exocytosis of the fungal cells from macrophages (39). This was due to oleic acid supplementation promoting an accumulation of actin around the yeast-containing phagosome within the macrophage (39). Formation of actin cups is an essential step in non-lytic exocytosis (78), since the process requires significant cytoskeletal remodeling. Indeed, MARCO has been shown to be a key regulator of actin-dependent cytoskeletal rearrangements in dendritic cells and microglia (79) and may therefore exert its negative regulator effects on this process via these functions, although MARCO has also been shown to regulate lipotoxicity in adipose macrophages and therefore may also be involved by maintaining healthy lipid homeostasis and recycling within macrophages (80). Another possible lipid-dependent mechanism of non-lytic exocytosis may involve type 1 interferon signaling and lipid droplets. Type 1 interferons are produced during viral infections and are an essential component of antiviral immune responses. Type 1 interferon signaling promotes non-lytic exocytosis of *C. neoformans*, since blocking IFN signaling abrogates the effect in cultured macrophages (81). This is particularly important to consider given that the majority of patients with cryptococcal meningitis are additionally infected with HIV. Therefore, the impact of activation of antiviral immune responses and interferon signaling may significantly affect subsequent antifungal immune responses, and this may correlate with viral and/or IFN-dependent remodeling of the lipid compartment in cells. Several viruses cause the increased formation of lipid droplets during infection, and this, in turn, helps to boost type 1 interferon signaling and supports protective immunity against viral infection (82). HIV infection of monocytes and macrophages significantly disrupts cholesterol recycling, resulting in the formation of lipid droplets due to reduced cholesterol efflux via ABCA1 (83). Whether these HIV-induced defects in lipid metabolism impact on the ability of macrophages to interact with *C. neoformans* or affect processes, such as non-lytic exocytosis, is not yet known. Yet, these will be important questions for the field going forward if we are to better ascertain how antifungal immunity is impacted by viral co-infections. Indeed, a better understanding of how viral and fungal co-infections have significant implications outside of *C. neoformans* infections is important, as several viral infections have now been identified as risk factors for development and/or worse outcome of invasive fungal infections, including *Aspergillus* infections in influenza patients, and mucormycosis in patients with severe COVID-19 (84).

### Macrophage functional polarization

Functional polarization of macrophages denotes the description of activation states that are either strongly pro-inflammatory (typically activated by lipopolysaccharide [LPS] and/or IFNγ), which is called “classic” activation or “M1,” or macrophages that are more anti-inflammatory and possess wound healing properties (typically activated by interleukin 4 [IL-4]), which is called “alternative” activation or “M2.” While these polarization states are readily observed in cultured macrophages *in vitro*, it is now accepted that these designations are probably an over-simplification of what occurs during immune responses in tissues and that macrophages likely exist on a spectrum of activation states that change depending on the tissue type and ontogeny of the macrophage. Nonetheless, macrophages infected with *C. neoformans* often exhibit a strong polarization toward the “M2” phenotype both *in vitro* and *in vivo* (33), and this has also been observed in human tissues particularly within the brain (85). As discussed earlier, counteracting this M2-like polarization of macrophages and, instead, promoting an M1-like functional profile (via CD4 T-cell derived IFNγ) is considered to promote protection against *C. neoformans* infection. There is therefore great interest in how macrophage functional polarization is regulated in the context of *C. neoformans* infection.

Macrophage functional polarization is intimately linked with lipid homeostasis and remodeling. Lipidome analysis of M1- and M2-activated THP-1 macrophages *in vitro* showed that M1-polarized macrophages had greater abundance of the mitochondria-localized lipid phosphatidylglycerol (PG), while M2-polarized macrophages had a significant upregulation of lyso-glycerophospholipids (GPLs) and phosphatidylinositol (PI) lipid species (2). Interestingly, some PI lipids have been shown to modulate antimicrobial functions of macrophages, rapidly increasing upon macrophage activation and boosting their production of reactive oxygen species (ROS) (86). Other studies have shown that stimulation of macrophages with LPS (to drive an M1-like activation) results in the upregulation of multiple antimicrobial proteins that accumulated on the surface of lipid droplets (87). These protein–lipid droplet complexes were found to act as innate immune signaling hubs, helping to build a scaffold for antimicrobial proteins and enabling the killing of intracellular pathogens (87). Whether a similar phenomenon occurs in fungal-infected macrophages is not known, but is interesting to consider in the context of *C. neoformans* infection since this fungus can feed off lipid droplets (39), yet is more efficiently killed by IFNγ-stimulated macrophages. It is likely that the nature of lipid droplets will differ in macrophages depending on their activation status, and this may inform in the ability of lipid droplets to act as immune signaling hubs. However, the heterogeneity of lipid droplets and the impact of this on innate immune responses is still poorly understood.

In addition to the changing lipid profiles of M1- or M2-activated macrophages, there is some evidence to suggest that signaling lipids may also influence macrophage polarization. As outlined earlier, S1P binding to S1P receptors regulates macrophage function and uptake of *C. neoformans*. Other studies have indicated that S1P stimulation of macrophages may promote an inflammatory phenotype; however, this appears to be context dependent. For example, in cultured primary macrophages stimulated with LPS, S1P induced a dose-dependent increase in M2 markers in these cells, driving the macrophages toward an M2-like phenotype in an IL-4-dependent manner (88). In contrast, a mouse model of lupus nephritis showed that S1P signaling via S1PR1 drove M1 polarization of kidney macrophages, which was accompanied by inflammasome activation and inflammatory tissue damage (89).

In the context of fungal infection, there is little research completed to date that examines how lipid remodeling may contribute toward macrophage functional phenotypes and antifungal immunity. There have been some recent advances made in other fields, however, which provide some insights into how lipid metabolism regulates macrophage functional phenotypes that are relevant for anti-*C*. *neoformans* immunity. For example, tumor-associated macrophages also exhibit a strong M2-like bias similar to *C. neoformans*-infected macrophages. In a mouse model of ovarian cancer, tumor-associated macrophages were found to upregulate genes involved with cholesterol recycling and efflux via ABCA1. The increased export of cholesterol from tumor-associated macrophages led to loss of lipid rafts that, in turn, promoted IL-4 signaling. IL-4 promotes the M2-like activation phenotype, which drives an anti-inflammatory environment that is pro-tumorigenic (90). It will therefore be interesting to determine if similar metabolic pathways that regulate cholesterol content of macrophages occur during fungal infection and if this contributes toward the phenotypes that support intracellular infection and limit fungal killing.

### The respiratory burst and production of reactive nitrogen species

To kill microbes following phagocytosis, macrophages will rapidly increase the production of ROS in a process called the respiratory burst, as well as increase production of reactive nitrogen species (RNS). Enzyme NADPH oxidase assembles in the phagolysosome membrane and initiates the production of progressively more toxic variations of ROS via a cascade of enzymatic reactions. Expression of inducible enzymes, such as iNOS, is activated downstream of IFNγ stimulation and drive the production of nitric oxide from arginine. Both ROS and RNS detrimentally damage the DNA, proteins, and lipids of microbial cells within a phagolysosome and therefore play a crucial role in the clearance of invading fungal pathogens, including *C. neoformans* (91).

Various studies have now linked the dysregulation of lipid metabolism with altered production of both ROS and RNS, contributing toward pathology. For example, macrophages with an over-abundance of cholesterol have been shown to excessively produce ROS/RNS, linked with increased expression of NADPH oxidase and iNOS. Macrophages that are laden with cholesterol are called “foamy” macrophages due to the soap bubble-type appearance of their cytoplasm, caused by the accumulation of lipid droplets inside the cell. Foamy macrophages are commonly observed in conditions, such as atherosclerosis, but have also been observed to form during some infections and in tissues with a high lipid content (4). However, there is some discrepancies in the literature regarding the inflammatory status of foamy macrophages and how this might affect production of ROS/RNS and subsequent pathology. Macrophage-specific deletion of cholesterol efflux transporter ABCA1 resulted in significantly increased free cholesterol content and lipid rafts in the plasma membrane following LPS stimulation, and this correlated with enhanced expression of iNOS (92). However, increasing cholesterol content of macrophages via other methods can have the opposing effect. Macrophage cell lines given exogenous cholesterol treatment induced an intracellular accumulation of cholesterol, but this was found to decrease the production of ROS and RNS (93). This may reflect different strategies employed by macrophages to regulate their cholesterol content that are influenced by external stimuli (e.g., presence of LPS) and crosstalk between receptors, which affect expression of enzymes required for cholesterol packaging and export from the cell. During fungal infection, the relationship between lipid remodeling and activation of fungal killing pathways is still unclear. However, it will be important to determine these relationships in relevant macrophage subsets, as this will have a significant influence on the pathways involved. For example, alveolar macrophages in the lungs are uniquely equipped to uptake and clear dying cells and are therefore specialized at dealing with excess cholesterol (4), whereas this may be less true for lung interstitial macrophage subsets that are more susceptible to intracellular infection with *C. neoformans* (33). Whether the lipid micro-environment and/or differences in lipid remodeling that occur in these cell types during infection are able to influence these phenotypes is not yet known, but represents an important future direction in developing a better understanding of the *C. neoformans* killing mechanisms used by macrophages.

### Inflammasome activation

A major inflammatory function of macrophages that is intimately linked with lipid homeostasis in macrophages is activation of the inflammasome. The inflammasome is a multi-protein complex that forms following a two-signal activation process, which (i) increases expression of inactive inflammasome components (e.g., pro-IL-1β, pro-caspase-1) and (ii) causes the formation of proteolytic cytosolic complexes that process inactive cytokines into active cytokines (IL-1β, IL-18) that can be exported from the cell via Gasdermin-D pores. Inflammasomes are activated in response to damage signals and/or microbial stimulation, but may also activate during sterile inflammation and are associated with multiple inflammatory disorders (94). The canonical NLRP3 inflammasome is the best-studied inflammasome and has been shown to promote antifungal immune responses to *Aspergillus* and *Candida* species (95, 96). During fungal infection, inflammasome assembly and activation mediates pyroptosis, a specialized form of inflammatory cell death, which can aid escape of fungal pathogens from macrophages (97). The capsule of *C. neoformans* appears to inhibit inflammasome activation in macrophages (98), with other studies showing that secreted fungal components may even actively inhibit assembly of the NLRP3 inflammasome (99). The role of IL-1β and NLRP3 in control of *C. neoformans* infection is therefore less likely to be directly relevant in fungal killing by macrophages. However, there is evidence that dysregulation of lipids can stimulate inflammasome activation and IL-1β secretion by macrophages, and this can have significant consequences on the functioning of the blood–brain barrier (BBB) and therefore potentially fungal CNS invasion.

Increased cholesterol content and exogenous exposure to cholesterol enhances macrophage inflammasome activation. Treatment of cultured macrophages with cholesterol crystals resulted in rapid phagocytosis of the crystals, which were stored within lipid droplets. These cholesterol-laden lipid droplets subsequently drove a dose-dependent secretion of IL-1β, which was dependent on caspase-1 and NLRP3 (100). Later work showed that this was dependent on intracellular complement C5ar1 signaling, whereby the complement component C5a bound to receptors on mitochondria to drive ROS production and provide one of the required activating signals for inflammasome assembly and IL-1β production, specifically in response to sterile inflammation caused by exposure to cholesterol crystals (101).

Other evidence suggests that cholesterol-mediated activation of the inflammasome is linked to cellular stress, which is a significant upstream activator of the inflammasome, caused by the pressure put on the lipid recycling system by the excess cholesterol in the cell (3). The NLRP3 inflammasome and excess lipid droplet formation have been linked with increased permeability across the BBB (102), while treatment of brain endothelial cells with IL-1β reduces the expression of tight-junction proteins and impairs barrier function (103). Although *C. neoformans* does not appear to be a strong activator of the inflammasome pathway itself, HIV infection of monocytes can trigger inflammasome activation and IL-1β release (104). This may be linked with the disruption to cholesterol recycling caused by the viral infection (discussed above), although this has not yet been formally linked. During *Toxoplasma gondii* CNS infection, release of IL-1β from microglia acts on IL-1R expressed on cerebral blood vessels to mediate inflammation and immune cell recruitment (105). There are therefore several lines of evidence that IL-1β release in the CNS may affect permeability across the BBB, which may predispose or enhance fungal invasion into this tissue. Future work should aim to examine the lipidome changes that occur during *C. neoformans* brain infection, with an aim to determine whether these influence BBB function and fungal CNS invasion via processes such as inflammasome activation. These are important questions to tackle in the field moving forward, as we urgently require a deeper understanding of how *C. neoformans* invades the brain vasculature to be able to develop targeted therapies to prevent development of meningitis.

## FUTURE DIRECTIONS

Research into the dynamics of the lipidome in inflammatory immune cells is still in the early stages. Yet, current work has already revealed intriguing relationships between the regulation of lipid metabolism and leukocyte function. As outlined above, studies have shown that exogenous treatment of macrophages with various lipids may change their function and/or interrupt their interaction with fungi. Tools to track and study changes in lipid abundance and localization are still evolving, but these exciting new technologies are expected to deliver a clearer understanding of how immune responses are regulated and influenced by lipids. Importantly, there is emerging evidence that lipid-dependent influences on macrophage responses may be specific to macrophage subsets, which differ in their ontogeny and localization. This is particularly important to consider in the brain, where there are marked functional differences between parenchymal microglia populations and border macrophages (e.g., in the meninges), for which we currently have a limited understanding of their role in infection.

Invasive fungal infections remain a critical threat to vulnerable patients, and we urgently require novel approaches to treat and manage these infections. Although still poorly understood, a closer examination of the contribution of lipid metabolism to antifungal immunity and macrophage–fungal interactions may provide some exciting new avenues for exploration in future studies. Indeed, there is evidence that lipids not only affect macrophage function and phenotype, but that these host molecules may also influence the phenotype of pathogenic fungi. For example, drug-resistant *Aspergillus* species have been shown to utilize host lipids to protect against drug-induced membrane stress and mediate their resistance to drug exposure (106). It will therefore be interesting to determine how lipids influence how fungi are recognized and phagocytosed by innate immune cells, but also how these lipid changes affect the behavior of fungal cells while within the host. In summary, lipids exert a significant influence over macrophage function and innate immunity, which may have profound implications for macrophage–fungal interactions, and further developing this area of research promises a great wealth for future discovery.
